# Development of Sterile Insect Technique for Control of the European Grapevine Moth, *Lobesia botrana,* in Urban Areas of Chile

**DOI:** 10.3390/insects12050378

**Published:** 2021-04-22

**Authors:** Gregory. S. Simmons, Melissa Cristal Salazar Sepulveda, Edith Alejandra Fuentes Barrios, Marcela Idalsoaga Villegas, Raul Enrique Medina Jimenez, Alvaro Rodrigo Garrido Jerez, Ruth Henderson, Hernán Donoso Riffo

**Affiliations:** 1 United States Department of Agriculture, Animal Plant Health Inspection Service, Plant Protection and Quarantine, Science and Technology, Salinas, CA 93905, USA; Ruth.Henderson@usda.gov; 2 Servicio Agrícola y Ganadero, Santiago 8330336, Chile; marcela.idalsoaga@sag.gob.cl (M.I.V.); raul.medina@sag.gob.cl (R.E.M.J.); Alvaro.Garrido@sag.gob.cl (A.R.G.J.); hernan.donoso@sag.gob.cl (H.D.R.)

**Keywords:** grape pests, invasive pests, mass-rearing, artificial diets, area-wide control

## Abstract

**Simple Summary:**

The establishment of the European grapevine moth in Chile presented significant production and export concerns for the grape and fruit industries. A national control campaign was launched in response. Infestations in urban areas adjacent to agricultural production areas were a significant challenge for control due to the difficulties in mounting effective measures in residential areas. The Servicio Agrícola y Ganadero launched a program to develop a sterile insect technique (SIT) as a means to provide an environmentally friendly method of control in areas where other control measures were not possible to employ. Here, we report the progress made to develop the SIT response capacity with a production of 75,000 sterile moths per week, as well as the results from a season-long SIT release program in a section of a small city in a grape and fruit production region in central Chile. Because of the high moth population in this area, the release of sterile moths did not reach large enough ratios of sterile to wild moths to achieve effective control, but high moth quality and field performance were observed. Recommendations are provided for further development of SIT and how to integrate its use into an effective area-wide control program.

**Abstract:**

The European grapevine moth, a Palearctic pest, was first detected in the Americas in 2008. Its establishment in Chile presented production and export issues for grapes and other fruits, and a national control campaign was launched. Urban areas next to agricultural production areas were recognized as a challenge for effective control. In 2015, a SIT laboratory was established in Arica, Chile to evaluate its potential for urban control. Progress included the development and evaluation of artificial diets, a mass-rearing of 75,000 moths/week, confirmation of 150 Gy as an operational dose for inherited sterility, and releases of sterile moths in a 25 ha urban area next to fruit production areas. Season-long releases demonstrated that high overflooding ratios were achieved early in the season but decreased with a large increase in the wild moth population. Sterile moth quality was consistently high, and moths were observed living in the field up to 10 days and dispersing up to 800 m. Recommendations for further development of the SIT include conducting cage and field studies to evaluate overflooding ratios and mating competitiveness, measuring of infestation densities in release and no-release areas, and conducting trials to evaluate combining SIT with compatible integrated pest management (IPM) tactics such as fruit stripping and use of mating disruption.

## 1. Introduction

The European grapevine moth (EGVM), *Lobesia botrana* (Denis & Schiffermüller), is a tortricid moth that has historically been a pest in the Mediterranean regions of Europe, North Africa, and Asia. Recently, it was introduced into the Americas with first detections in Chile in 2008, California, USA in 2009, and Argentina in 2010 [1,2]. Grapevine flowers and berries are favored hosts for the EGVM. Other hosts include olive flowers, blueberries, and plums.

The EGVM has multiple generations a year, starting in the spring from overwintering pupae with 3–4 generations observed in Mediterranean areas and three generations documented in California, Chile, and Argentina. In the Americas, the third generation goes into winter diapause as pupae with reports from California of the occurrence of a portion of the second generation going into diapause [1,2,3,4,5]. After overwintering, adults emerge and start the first generation by ovipositing on flowers. Larvae hatch and form a feeding nest by webbing together several flowers. Successive generations target developing stages of grapes, with the first generation feeding on flower clusters, the second generation on green berries, and the third generation inside the bunches of ripe grapes after veraison, i.e., the change in color of the grape berries. Webbing within the fruit clusters may be apparent, along with excrement and shriveled berries. Feeding on berries causes direct damage and leads to fungal infections that can cause extensive rot, resulting in bad flavors in wine, making heavily infested grapes unusable and leading to a total loss of clusters [2,6].

In April of 2008, the first confirmed detection of EGVM on the South American continent was made in the Linderos area of Maipo province in the Metropolitan region in Chile [7]. Based on EGVM’s status as a significant grape pest in other parts of the world, its establishment in Chile presented significant production and export issues for grapes, as well as for other fresh market agricultural commodities. In response to this EGVM detection, the Chilean agricultural authority, the Servicio Agrícola y Ganadero (SAG), declared EGVM under official control and started working in a cooperative effort with the Chilean grape and fruit industries and university scientists to mount a national campaign to control EGVM [2,7,8,9]. Control operations included a monitoring program for all grape-growing regions (both wine and table grapes), coordinated applications of pesticides and mating disruption, and regulatory control activities to limit the spread of infested fruit and other commodities [8,9,10]. Over time, it became clear that urban areas close to grape production areas and fruit production remained a significant risk for controlling EGVM. This was due to the popularity of growing grapes in Chilean home gardens and the difficulty of applying coordinated environmentally friendly control measures against pests in urban environments. Significant areas of grape and fruit production in regions to the south of Santiago are in close proximity to small cities and residential areas that can harbor EGVM on home garden grapevines [2,7]. After these first years of the infestation, there were detections in fruits other than grapes such as plums and blueberries. While these fruits are not typically preferred hosts of EGVM, they can at times be attacked, particularly when there are infestations in grapevines nearby and some spillover can occur [2].

Because of detections in blueberries, export requirements have been put in place to allow continued market access. These requirements include the operation of preclearance inspection and systems approaches for production and packing of fruit from areas of low pest prevalence and fumigation treatments from generally infested areas [11,12]. While these measures are effective, they are costly to implement. It is in areas where residential areas with abundant EGVM host plants are near to grape and fruit production that are at greatest risk of ongoing impacts of EGVM infestation and are also more challenging to control because of the limitations of mounting effective control measures in urban environments [2,7].

To address these concerns, in 2016, SAG, with cooperation from the fruit industry and the Chilean Atomic Energy Commission, began investigating the use of the sterile insect technique (SIT) as a strategy for control of EGVM in urban areas. This work was supported by a technical cooperation agreement with the FAO/IAEA, which funded training for Chilean technical staff, sponsored expert missions from member countries, and supplied key materials and equipment [7,13,14]. The program began in 2015 with the establishment of an EGVM SIT laboratory in Arica dedicated to developing mass-rearing methods and conducting radiation biology studies, with parallel work by the lead fruit industry research organization at their laboratory in Santiago (Fundación para el Desarrollo Frutícola, FDF). With the successful development of a rearing system capable of producing tens of thousands of moths per week and completion of radiation biology studies, SAG launched the first year of a 2 to 3 year pilot project to evaluate the use of the sterile insect technique for control of EGVM in urban areas. Here, we report the progress made to develop a mass-production system for sterile EGVM and the results from the first year of a pilot program to carry out season-long releases in a 25 ha area of a small city in close proximity to grape and fruit production areas.

## 2. Development of Mass Rearing Program

The work to develop the rearing system relied on adapting methods developed from moth SIT programs for the codling moth, *Cydia pomonella* (L.), the pink bollworm, *Pectinophora gossypiella* (Saunders), and the light brown apple moth, *Epiphyas postvittana* (Walker, 1863) (see [15]). Similar to rearing systems for the codling moth and other tortricid moth species, EGVM larvae pupate in the diet and are difficult to separate easily. Therefore, these rearing systems rely on the collection of adults straight from relatively shallow trays of diet [16]. The female moths prefer to lay eggs on smooth plastic or wax-covered paper surfaces, often in folds or creases in the oviposition substrate material. Taking rearing methodology insights from these other programs, the work in Arica progressed focusing on adapting these methods for the development of a mass-rearing system for EGVM.

### 2.1. Development of Larval Rearing Diets

Following the successful rearing of EGVM in USDA laboratories on the pink bollworm diet [17], the Chilean program started by adapting this diet for the mass-rearing program. An extensive period development was conducted over about 3 years to adapt the diet to more readily available ingredients in Chile. During this period, 46 diet formulations were evaluated, which involved testing varying formulations of soy flour, wheat germ, sawdust, vitamins, agar, antibiotics, and preservatives. Part of this work included evaluation and modification of diet used for the production of sterile Mediterranean fruit fly, *Ceratitis capitata* (Wiedemann), in the SAG Moscamed production facility in Arica. This resulted in the incorporation of sawdust to provide a bulking agent which allowed the elimination and reduction of the more costly ingredients, alphacel and agar, present in the pink bollworm diet.

Several diets achieved satisfactory results as measured by increased pupal weight, higher pupal eclosion rates, and higher numbers of adults harvested per unit of diet. Of the best performing diets, #44 and #46 produced pupae with the highest average weights (average weight of combined male and female pupae) of 9.7 and 9.0 mg, respectively, and with eclosion rates exceeding 90%.

Diet #46 is the current production diet, with the main difference from other similarly performing diets being the addition of the locally sourced Vitamínico mix to replace the more costly Vanderzant’ s vitamin mix (Table 1). It has one of the higher harvest rates ranging from 1600 to 2400 pupae per kg of diet. This production figure compares well to other EGVM rearing systems using the pink bollworm diet and to rearing systems for another tortricid, the light brown apple moth, which can reach yields of up to 1200 to 1500 pupae per kg of diet.

For the current moth production levels, two batches of 44 kg of diet #46 are made per week. The process is started by adding agar to the water and mixing until dissolved and boiling for 3 min and then allowed to cool to 45 °C. Once the agar is cooled, the rest of the liquid ingredients (10% formaldehyde and 25% acetic acid solutions and corn oil) are mixed in. All of the dry ingredients are mixed together and added to the agar mixture, and all contents are blended in a 60 L commercial bakery mixer for ~15 min. Before incorporation into the diet the wheat germ, and sawdust are sterilized at 100 °C in a dry oven for 1 h.

### 2.2. Oviposition Cages

**Figure 1:**
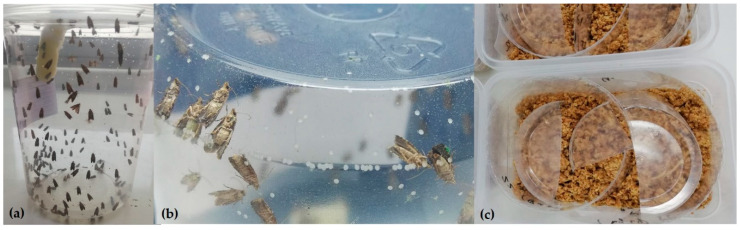
(**a**) One liter plastic oviposition cages with cotton wick for adult feeding. (**b**) Female moths oviposit directly onto plastic surface. (**c**) Larval rearing tray infested with EGVM eggs on cut plastic strips.

Oviposition cages are 1 L clear plastic cups with lids fitted with a honey water cotton wick (Figure 1). Sugar has been used, but better results are obtained with honey due to fewer problems with mold growth. Preservatives are not added to the honey or sugar solution. Cages are set up with 170 adults in a 1.5:1 male sex ratio collected from the collection system and maintained at 22 ± 1 °C, 65 ± 5% RH, and a light–dark (LD) 16:8 h photoperiod. Female moths oviposit on the sides, top, and bottom of the cup. Eggs are collected every 3 days (2–3 times over the life of the cage) for up to 9 days. Eggs are collected by cutting the plastic cup into strips for infesting. Moths are transferred to a new cage for a second and third egg collection. Egg harvests range from 1500 to 2000 eggs per cage.

Harvested eggs are cut into four strips of approximately equal numbers of eggs and treated in a 1.3% NaOH solution for 3 min to sterilize, before being allowed to dry, and they are then used to infest rearing trays on the same day.

### 2.3. Larval Rearing

A 2 L rectangular plastic rearing tray (14.5 cm × 21 cm × 9 cm) is filled with 0.4 kg of diet to a depth of about 4–5 cm. The tray is covered with a Tyvek™ paper top, which allows moisture and gases to pass, and it is fitted with a snap cover cut with two 2.5 cm diameter holes for ventilation. Trays are infested four times per week with eggs that vary from 1 day to 3 days old with 1500 to 2000 eggs per tray. Trays are infested by setting cut egg strips in the diet on the edge such that eggs do not rest directly on the diet, which can impact eclosion when plastic oviposition substrates are used (Figure 1).

Larval rearing conditions are maintained at a temperature of 24 ± 2 °C, 65 ± 5% RH, with a 24 h light cycle. The completion of the life cycle under these conditions averages 35 days from diet inoculation with eggs to the beginning of adult emergence. A light cycle of continuous 24 h light was chosen to stimulate faster larval development and to ensure that diapause is not initiated (see [18]).

### 2.4. Moth Collection

**Figure 2:**
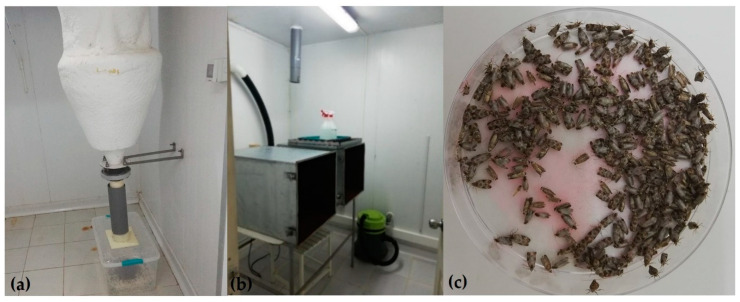
(**a**) Cyclone collection trap mounted in a cold room with collection box to hold moths immobilized by the cold. (**b**) Moth eclosion cabinets fitted with a fiber optic UV light and PVC ductwork vacuum system leading to the cyclone trap. (**c**) Irradiated EGVM moths marked with Dayglo^TM^ fluorescent powder in Petri plate for release.

A small-scale adult collection system adapted from systems used by codling moth and pink bollworm sterile insect production facilities was installed for collection of adults directly from larval rearing trays, which is the method used in codling moth rearing [16]. The system consists of two sheet metal eclosion cabinets with a wide opening fitted with a cover at one end and a funnel shaped opening at the other end that measures 183 cm × 122 cm × 61 cm. Interior brackets are designed to hold the larval rearing trays. The interiors of the cabinets are lightproof, fitted with a UV fiber optic light placed inside a 63.5 mm diameter PVC pipe, and connected to the narrow opening at the funnel-shaped end. The light serves to attract moths into the PVC ductwork connected to a 1/3 hp (248.6 W) blower fan to create suction (~15.5 m^3^/s) to draw moths into a cyclone collection trap. The cyclone trap is mounted in a cold room maintained at 3 ± 2 °C, which immobilizes moths on entry, causing them to drop into a plastic collection box (Figure 2). The operation of the cyclone trap separates the lighter loose moth scales from the heavier adults that drop into a collection bucket, while scales are collected into a vacuum filter bag.

A total of 160 trays are loaded into an eclosion cabinet 35 days after egg infestation, and emergence begins within a day of loading. Trays are removed after 7 days of eclosion and reloaded. Collected moths are transferred into Petri plates at ~300–330 moths per plate with a small dusting of fluorescent Dayglo^TM^ powder (Day-Glo Corp., Cleveland, OH, USA) added at the rate of 2 g/L moths (Figure 2). These are held in cold storage at 7 ± 1 °C for up to 3 days until irradiation, shipping, and release. The daily collection ranges between 5000 to 10,000 moths per day for up to 75,000 moths per week that are available for the release program.

### 2.5. Irradiation

Previous research suggested 150 Gy was an appropriate dose to achieve high levels of sterility in EGVM [19], and the program evaluated this dose for its own production in order to confirm that this would be an effective dose for an F_1_ release strategy of sterile EGVM for the Chilean program. During 2017–2018, a series of crosses were performed using the Arica laboratory colony. These included combinations of each irradiated sex crossed with the opposite fertile sex, as well as irradiated crosses of both sexes. F_1_ generation survivors of the irradiated male by fertile female parental crosses were also crossed to estimate the degree of inherited sterility (IS).

At 150 Gy, both irradiated females crossed with irradiated male crosses and irradiated females crossed with fertile males had high sterility levels and a high index of sterility (Table S1, Supplementary Materials). While 150 Gy was confirmed as an effective dose for sterility, an operational decision was made to increase the dose to 160 Gy. An analysis of sterility achieved for irradiation for 160 Gy is shown in (Table S2 Supplementary Materials). The complete methods and results for this work are reported in the Supplementary Materials (Confirmatory Irradiation Studies, Tables S1 and S2). Moths are irradiated twice a week with an exposure of 160 Gy using one of the Moscamed Gammacell 220 Nordion irradiators in Arica. Because of the collection and irradiation schedule, moth age varies between 1 and 3 days old when irradiated. Irradiation of up to 25,000 moths is achieved by irradiating multiple stacks of eight Petri plates, each filled with 300 moths. This takes about an hour of irradiation time. The time for irradiation is long, as the Gammacell irradiator used for the moth program is near the end of its source life. The dose rate is determined using the decay chart for the Gammacell 220 irradiator on the basis of prior dose mapping using Fricke dosimetry.

### 2.6. Quality Control

To maintain insect quality in the mass-rearing operations and to evaluate the effects of changes and improvements to rearing procedures, extensive data on all aspects of the production process are collected as an ongoing activity. For both regular production and evaluation of new diets, subsamples are collected from each production lot and include data on egg production per oviposition cage, the percentage of egg eclosion, the number of pupae harvested per tray, pupal weight, pupal deformities, and pupal eclosion rates. Laboratory flight tests are conducted by placing samples of collected adults inside 1.5 m × 1 m × 1 m screen cages to determine what percentage of moths fly from a Petri plate. Additional data collected for evaluation of diet formulations include determining the number of eggs produced per female moth and the developmental time in days from egg to adult. The better-performing diets have more rapid development times, which also correlate with higher pupal weights, an indication of a more efficient diet with good nutritional quality.

## 3. Field Release Studies

### 3.1. Dispersal Studies in 2018–2019

During the months of October through February in 2018–2019, five field releases were conducted in a large open field within the small city of Requinoa in the O’Higgins region to the south of Santiago in Central Chile, an area with extensive grape production. The objectives were to evaluate sterile EGVM dispersal and survival under field conditions as an assessment of the field performance and quality of the sterile moths produced by the Arica program and to obtain information that would help design future pilot-scale field testing and operational releases.

Each field test was conducted with a point release of either 2500 or 5000 sterile moths from the center of a 350 m^2^ trap grid in a 12.5 ha area with an array of 40 white plastic delta traps (11.5 cm × 20 cm × 20.8 cm) with sticky base inserts (20 cm × 19 cm) (Feromonas Chile LTDA, Renca, Chile) baited with 1 mg *Lobesia botrana* lures (E7,Z9-12:Ac) (Pherocon-EGVM, Trécé Inc. Adair, OK, USA). Traps were mounted on 1 m stakes distributed around the center of the release grid. Sterile moths were marked with fluorescent powder as described above. Releases were made at midday after arrival of an overnight shipment of moths from Arica. Traps were checked daily for 7 days after release with all sterile and wild insects counted. The sticky trap bases were replaced with new bases after each collection. In addition to the release studies, a simultaneous field survival test was conducted for each shipment. These tests consisted of placing a Petri dish with 250 adults inside of a 60 cm tall × 30 cm diameter mesh field cage that was hung at about 1.5 m off the ground from a tree in the project area. There were three replications of this experiment for each date. Cages were checked on a daily basis for 7 days, and the number of live and dead moths were recorded.

**Figure 3:**
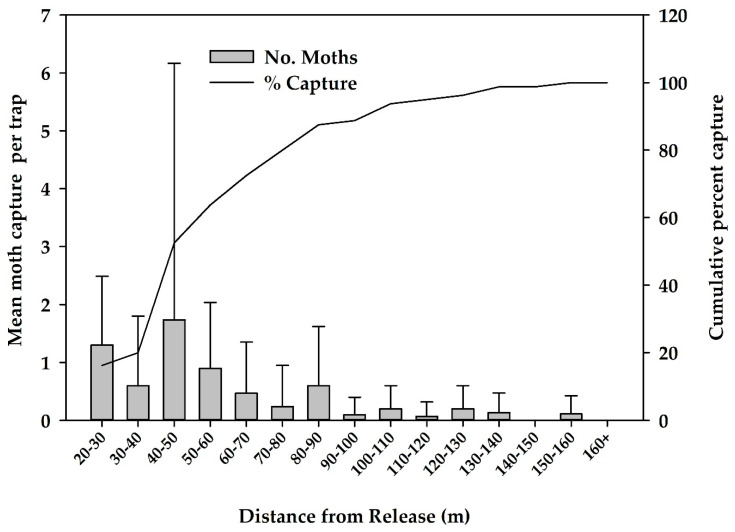
Mean (SD) number of sterile male moths caught at different distances after central point releases and the mean cumulative percentage of total recaptures in five sterile moth dispersal experiments October 2018 to February 2019 (*n* = 48 traps/experiment).

For the five experiments, there were a total of 80 male moths recaptured for a recapture rate of 0.7%. The mean (SD) dispersal distance of male moths was 58 m (28.2 m) with the farthest flight distance recorded of 155 m (Table 2, Figure 3). The mean (SD) survivorship of mixed sex samples of moths after 4 days was 34.4% (23.8%) declining to a mean (SD) of 1.0% (2.0%) by day 9 (Table 3).

### 3.2. Pilot Project Field Release Trials in 2019–2020

An operational-scale pilot field release project was started in 2019 to make season-long releases of sterile moths in an urban area next to a mixed agricultural crop region which included grape, berry, tree fruit, and mixed field crops. There were several project objectives. The first was to develop stable rearing, collection, irradiation, shipping, and release methods to advance the production system for sterile EGVM and help determine the feasibility of mounting a future season-long operational control program using sterile moths.

A second objective was to evaluate the performance of released sterile moths for use in an operational program and to obtain information about release rates, survivorship, and monitoring. Another key factor was to collect assessments of moth quality as it relates to the moth production system and the impacts of overnight transit in order to provide feedback needed to improve the overall system.

Last, it was also an opportunity to obtain detailed population data on the wild moth population in this area to determine the number of sterile moths and frequency of releases needed to control EGVM populations in urban areas, as well as what other IPM measures may be needed to complement the use of SIT.

#### 3.2.1. Sterile Moth Releases in Pilot Project

**Figure 4:**
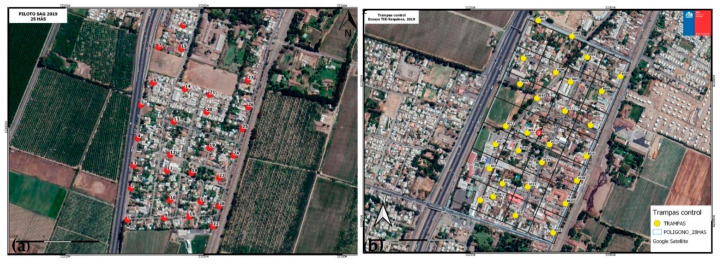
(**a**) Sterile EGVM release plot in Requinoa Chile, with red dots show monitoring trap locations (*n* = 34 traps). (**b**) Control no-release area to the north of the sterile release plot in Requinoa Chile, with yellow dots show monitoring trap locations (*n* = 28 traps).

**Figure 5:**
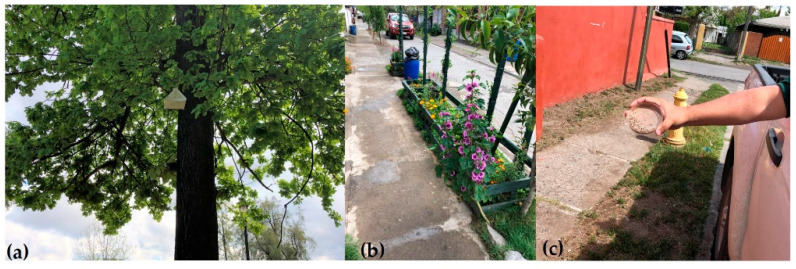
Requinoa release plot: (**a**) pheromone trap fixed to an ornamental tree; (**b**) a street along the release grid; (**c**) hand release of EGVM out the window of release vehicle driving at 2 km/h.

The pilot release area was a 25 ha grid in the southernmost section of the small city of Requinoa, a rectangular area of homes with small gardens and open spaces with residential surface streets with homes, parks, and a light commercial business area with light to moderate traffic (Figure 4 and Figure 5). The south, west, and east borders are adjacent to crop lands. The city continues past the northern border of the grid, where a similar sized grid was added about 100 m to the north to serve as a control no-release area (Figure 4). Each area was set up with a network of large plastic delta traps baited with pheromone, hung from ornamental non-host trees and, in some cases, inanimate objects such as fences and posts, at a height of 3–4 m. Trap locations varied from 80 to 130 m apart depending on the terrain and access to trap sites (Figure 5). In the release grid, there were 34 traps, while the control grid had a network consisting of 28 traps (Figure 4).

Increased production for the field program was started in July of 2019 to provide the releases, with a goal of releasing 50,000 moths per week for a release rate of 2000 moths/ha/week split into two releases of 25,000 moths each on Tuesday and Friday of each week (Table 4). Moths were collected 3 days per week for each release and kept in plastic Petri plates (100 mm × 15 mm) ranging from 282–378 moths/plate. These were held at 7 ± 1 °C until irradiation at 160 Gy in the afternoon before each release. Just before irradiation at 160 Gy, moths were dusted with colored fluorescent powder as described above. Irradiated moths were packed into an insulated foam ice chest with gel ice to hold at a target temperature of 7 °C, shipped by air overnight to Santiago, and transported to the field release site in Requinoa.

**Figure 6:**
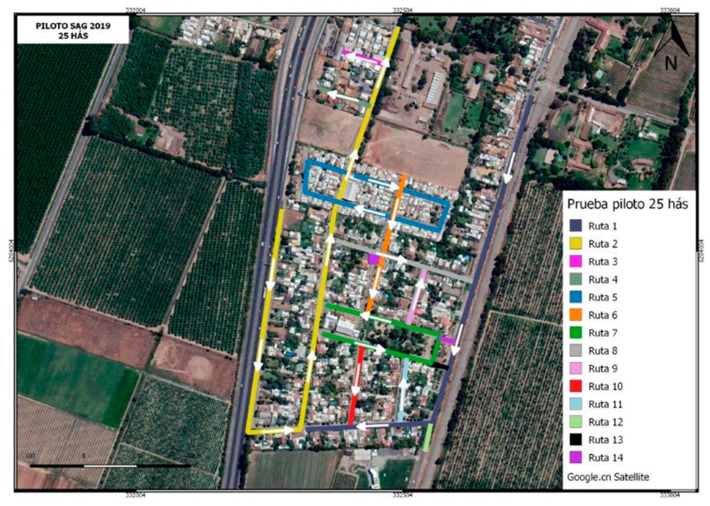
Sterile moth release route on city streets of Requinoa and aerial view of surrounding crop lands.

Moth shipments arrived at the field site at about 11:00 a.m., about 20 h post irradiation. Moths were released from the Petri plates out the window of a truck moving slowly at 2 km/h on a fixed grid pattern throughout the release area (Figure 5 and Figure 6). The number of plates available was divided by the number of street sections for each route so that a roughly equivalent number of moths were released in all sections of the grid. The releases took about 45 min to complete.

After the field release, three sample release Petri plates corresponding to each collection date were transported back to the Rancagua lab and placed in an outside sunny garden area to conduct a flight ability test for moths from each collection date within the release cohort. These moths were allowed to leave the Petri plates over a period of about 2 h. Moths that did not fly or died were counted as nonfliers, allowing the percentage of flyers to be calculated for each release.

The traps in the release and control plots were inspected once a week, while sticky base inserts were collected and replaced with new sticky bases. Sterile and wild moths on trap bases were counted in the laboratory and checked for the presence of the fluorescent dust using a handheld UV light to identify sterile from wild moths. Traps were checked on Monday of each week before the new weekly releases, and recapture rates were calculated as the number of moths caught divided by the number released in the previous week. Overflooding ratios were calculated by dividing the total number of sterile to wild moths for each trap and calculating the mean (SE) for all of the traps. Differences in wild moth trap catch were analyzed for significance by repeated-measures ANOVA (PROC GLM SAS^®^, Version 9.4. SAS Institute Inc., Cary, NC, USA, 1989–2019).

#### 3.2.2. Results of Sterile Moth Releases in Pilot Project

**Figure 7:**
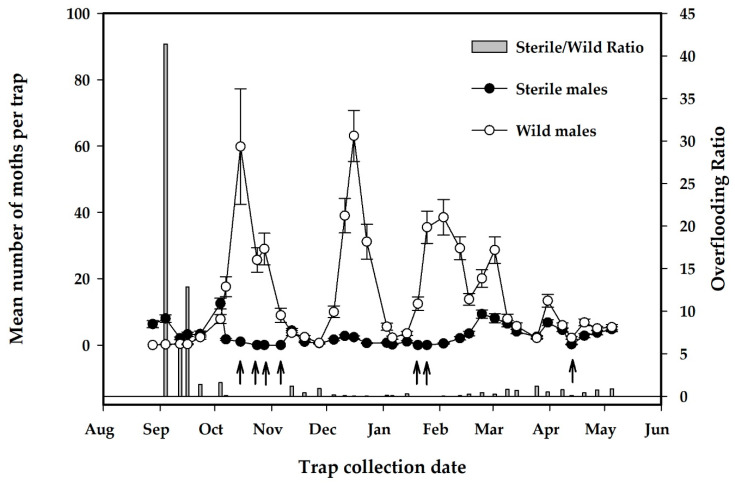
Mean number of sterile and wild moths per trap per week in release plot and ratio of sterile to wild moths (*n* = 34 traps). Error bars are standard errors of the mean. ↑ shows weeks without sterile release.

**Figure 8:**
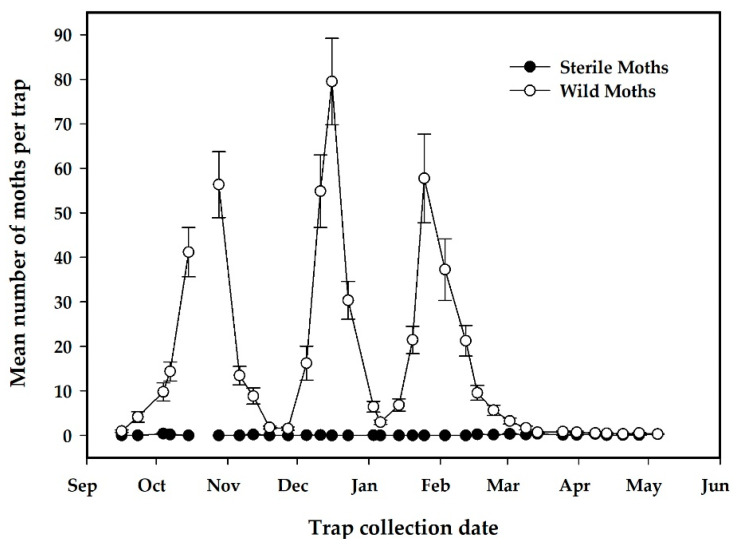
Mean number of sterile and wild moths per trap per week in no-release plot (*n* = 28 traps). Error bars are standard errors of the mean.

Project releases began on 20 August 2019 and continued for 37 weeks to 5 May 2020. There were seven complete weeks of missed releases and three other occasions where only one release was made per week (Figure 7), resulting in 57 releases, a total 752,353 released sterile EGVM and a mean (SE) release rate of 813 (108) moths/ha/week (Table 4). The percentage of flyers from the flight ability tests of release samples had a mean (SE) of 80.9% (2.8%) with a range of 21.9% to 93.5% over the course of 57 shipments (Table 4). For both the release and the control areas, there were three distinct flights of EGVM with the peak population of wild moths occurring in late December for both study areas (Figure 7 and Figure 8).

**Figure 9:**
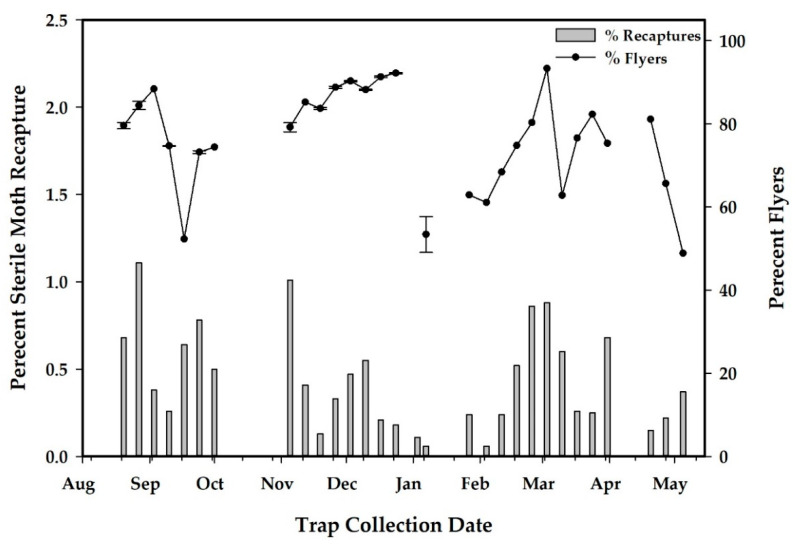
Percentage of sterile moths recaptured on pheromone traps in relation to the percentage of moths that flew in a field laboratory flight ability test (*n* = 34 traps). No relationship was observed between percentage recapture and the percentage of moths that flew (*F*_1,27_ = 0.39, *p* = 0.54).

Recapture rates estimated for sterile male moths were variable over the course of the experiment with a mean (SE) of 0.9% (0.1) and a range from 0.1% to 2.2% (Table 5). Recapture rates were highest during the first weeks of releases and were reduced in the middle period of releases. Data on the percentage recapture and percentage of moths that flew were analyzed by linear regression after arcsine transformation to normalize the data. This analysis shows no relationship between the percentage of recaptured sterile moths and the percentage of flyers (*F*_1,27_ = 0.39, *p* = 0.54) (Figure 9).

Male EGVM become active at dusk and start flying for the first few hours of the night responding to calling females or pheromone baited traps when temperatures exceed 12 °C [20,21]. To determine if evening temperatures had an effect on male moth recapture rate, the average weekly temperature at dusk was calculated by averaging the two hourly temperature readings before and after dusk (see [22]) for each day of the week. The percentage of recapture data were normalized using the arcsine transformation and analyzed by linear regression. The effect of daily high temperature on moth recapture rates was also analyzed by linear regression by regressing recaptured rates on the average daily maximum temperature.

**Figure 10:**
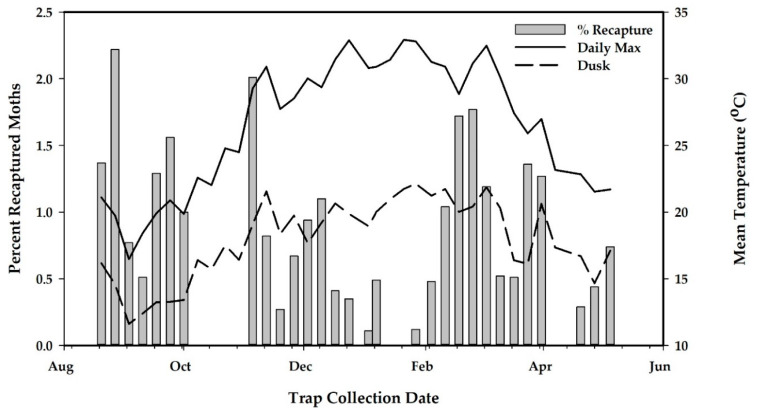
Percentage of sterile moths recaptured on pheromone traps in relation to weekly mean temperatures at dusk and the daily maximum temperatures (*n* = 34 traps). No relationship was observed between percentage recapture and average weekly temperature at dusk *F*_1,28_ = 0.89, *p* = 0.35) or between percentage recapture and average daily maximum temperature (*F*_1,28_ = 1.40, *p* = 0.35).

Linear regression analysis found no relationship between percentage recapture and average weekly temperature at dusk (*F*_1,28_ = 0.89, *p* = 0.35), as recapture rates were variable for several months when early evening temperatures were suitable for flying (Figure 10). As daytime temperatures increased to >30 °C, sterile moth recapture rates, while still variable, were at the lowest during this time period (Figure 10) although there was no relationship between percentage recapture and average daily maximum temperature (*F*_1,28_ = 1.40, *p* = 0.35).

The mean overflooding ratio of sterile to wild moths as measured on traps had a mean (SE) of 2.1 (1.2) with a range of 0 to 41.4 (Table 5). For the first 6 weeks of releases, overflooding ratios were higher when wild moth captures were lower. Increasing wild moth populations and some missed releases contributed to this ratio decreasing to much lower levels for the remainder of the experiment (Figure 8).

A lower number of wild moths was seen in the release field compared to the control field, which may have been an effect of high overflooding ratios achieved by the early releases on the growth rate of the wild moth population, as peak numbers of moths per trap for the second and third flights were lower in the release plot than the no-release plots (Figure 8 and Figure 9). The number of wild moths captured per trap for the entire monitoring period for the release field had a mean (SE) of 14.7 (0.6) vs. 15.7 (0.7) for the control field. For statistical analysis, these data were transformed with a log transformation to normalize data followed by a paired *t*-test and not found to be significantly different (*t*(68) = 0.44, *p* = 0.66). As a means to determine if there may have been a significant effect of migration from surrounding areas on the wild moth population capture in the release and control fields, an additional analysis of the spatial distribution of the mean wild EGVM trap catch by location for the release and control field is shown in the Supplementary Materials (Spatial Analysis of Wild EGVM Trap Captures in Release and Control Plots, Figure S1).

**Figure 11:**
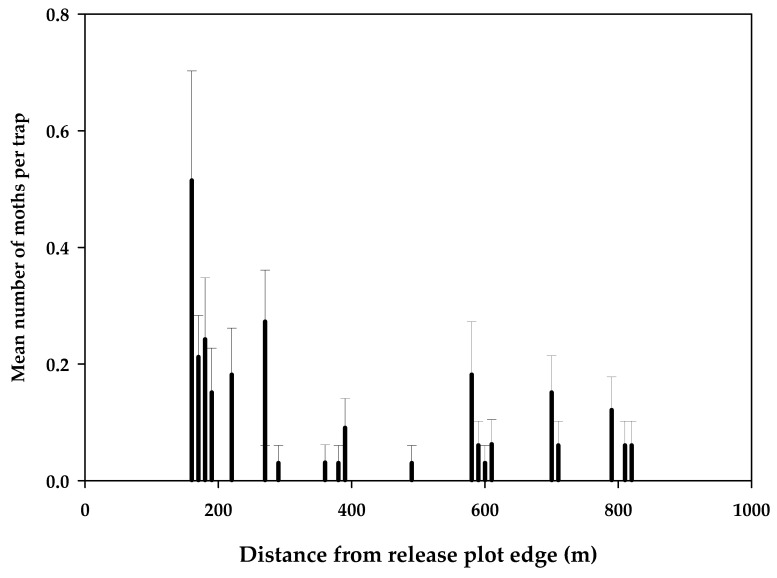
Mean number of sterile EGVM caught per trap in the no-release control field as measured from the nearest release plot edge over the entire release period of August 2019–May 2020 (*n* = 28 traps). Error bars are standard errors of the mean.

A total of 86 sterile moths were caught in the no-release plot over the course of the experiment which was 2.5% of the total (Table 5). The minimum distance these moths must have flown from the release area was estimated using the Google Earth measurement tool to determine the distance from the nearest northern edge of the release plot to a trap. Of the total recaptures, 21% were caught at 160 m from the release plot with 5% caught at greater than 800 m (Figure 11).

## 4. Discussion

Since 2015, with the opening of the EGVM sterile insect laboratory in Arica, there has been a sustained effort to develop mass-rearing methods for EGVM. This includes development and evaluation of 46 formulations of artificial diets which led to the selection of a high-performing diet made from lower cost ingredients available locally. The development of efficient larval rearing methods and an adult collection system demonstrated the capability of producing a stable supply of up to 75,000 moths per week. The results of radiation biology by other workers were evaluated with a thorough series of testing to confirm the earlier work and to develop an operational protocol to collect, store, irradiate, and ship sterile moths in suitable numbers for field testing.

The results for irradiation at 150 and 160 Gy were compared to results obtained by other workers [19,23] with similar levels of irradiated male fertility when outbred (irradiated × fertile) and low levels of fertility in outbred irradiated females. Although Saour (2014) reported 0% fertility for the irradiated female when outbred at 150 Gy [19], we found a small level of residual fertility of 4.1% and 3.4% for this cross at 150 and 160 Gy, respectively (Tables S1 and S2, Supplementary Materials). Steinitz et al. (2015) [23] also found a small amount of residual fertility at 150 Gy in irradiated EGVM females when outbred. Outbred males irradiated at 150 Gy had higher levels of fertility than Saour (2014) reported [19] but had similar sterility levels in the F_1_ generation.

The goal of setting the dose for use in an inherited sterility (IS) control strategy is to find the dose when outbred irradiated females are almost completely sterile, leaving irradiated males partially sterile with high sterility in their progeny [24,25,26]. The results reported here raises the question of how much residual fertility can be allowed for irradiated female EGVM and whether a range of 3–4% fertility meets this standard. Barclay (2001) [27] modeled incomplete sterility in sterile release programs and found that, at a threshold level of ~8% residual fertility, there would be little effect on program outcomes. The 3–4% residual fertility for outbred irradiated female EGVM found in this study is well below this threshold. Even for the condition of moderate residual fertility, the integration of other control methods with sterile releases will compensate for and still allow suppression of the population [27]. Kean et al. (2008) [28] modeled the effects of IS in SIT programs and showed that there is a risk of generating a self-sustaining irradiated lineage population if the irradiation dose is below a critical threshold value. They argued that release programs employing IS will be effective only if matings between irradiated lineage partners are not viable and added that fertility from matings in the irradiated lineage is rarely examined experimentally [28]. In the case of EGVM, we are fortunate that Steinitz et al. (2015) [23] examined fertility in F_2_ lineages from irradiated female parental crosses, which resulted in few offspring, and their expected contribution to a second-generation field population is marginal.

On the basis of the above analysis, we think that the current sterility levels in irradiated females at a dose of 150–160 Gy are acceptable for the current program goal to use an SIT IS strategy to suppress EGVM infesting urban areas. However, this may not be an acceptable sterility level when sterile moths are released to suppress or prevent EGVM from attacking grape and fruit crops, as partially fertile females moths could cause some level of damage to fruit and should be evaluated [24]. For this reason, testing the dose to achieve 100% sterility of irradiated female EGVM will be important for future development of options for use of the SIT to protect crops.

A field dispersal study in 2018–2019 demonstrated that, on average, ~62% of male moths irradiated at 150 Gy were recaptured within a 40–60 m range and the longest flight was 155 m (Figure 3). For three of the five releases, recapture rates were poor; therefore, these results contributed little to calculations of the mean dispersal distances. Except for the small accumulation of about 1.3 cm of rain during the second release replicate, the weather was dry during the entire period of the release experiment. The average temperature ranged from 14 to 21 °C during the release periods, well above the threshold temperature for flight of 12 °C at dusk [21]. It is possible that there were some quality differences between different cohorts of moths used for these releases that affected flight and recapture rates. These dispersal distances were greater than those observed by Saour (2016) [29], where 40 m was the farthest dispersal distance observed by male EGVM irradiated at 150 Gy but the farthest trap from the release point in Saour (2016) was only 50 m; thus, it is possible sterile moths flew out of the grid. We observed much longer distance dispersal in the 2019–2020 sterile release field trial with some sterile moths flying as far as 800 m (Figure 11). Male average male moth survivorship after 3 days was ~62%, declining to 1% by day 9 (Table 3).

An effort to track moth field longevity by using different colored dyes for each release week was unsuccessful due to an inability to distinguish between different colored dyes under an inspection protocol using handheld UV light. However, due to periods where there were cessations in release, it was possible to track moth longevity by observing moth captures in weeks when no releases were made. There were three periods of sterile moth captures after cessation of releases where 60, eight, and seven marked sterile moths were recaptured after periods of 6, 10, and 14 days, respectively.

Taken together with the results from the dispersal experiment in 2018, many sterile moths were active for at least 6 days after release, with evidence that some moths were present in the release environment and responded to pheromone for as long as 14 days. However, we could not calculate percentage survivorship for any of the release cohorts from the 2019–2020 study. We instead used the 2018–2019 study results, which showed that about half of the male moths dispersed within an area of 60 m, suggesting that setting a distance between release gridlines of about 100 m apart would allow for a more even distribution of dispersed moths throughout the area for ground release. Moreover, with an average survivorship of 62% after 3 days, a release frequency of twice per week would maintain sterile moths at about a constant population level.

The program conducted a season-long field test from August 2019 to May of 2020, establishing two 25 ha areas for a release and no-release control area within a city environment. The program made a total of 57 releases, resulting in a total release of 750,000 sterile moths, and conducted 37 weeks of weekly monitoring. Results from this study showed that, in the first month in the early spring, high overflooding ratios were achieved but these declined as wild EGVM populations increased. The period of the lowest recapture rates corresponded to the middle of the release season when daily temperatures were highest (Figure 10). While there was no significant relationship between recapture rates and higher temperatures, as releases were made at about 11:00 a.m. each day, released moths would experience the heat of the afternoon, which may have had an impact on their performance or survivability on their first night of flying.

There was also no relationship between the percentage of moths that flew in flight ability tests and recapture rates, although there were some observed declines in the percentage of flyers for some release cohorts during the warmest days of the trial. This may be a case where a moth quality factor interacted with high heat to impact recapture rates. It is not known why, for some release cohorts, there were declines in the average percentage of flyers to as low as 50% on some dates. For some shipments, shipping temperatures were not well controlled at the 7 °C target and, on some occasions, fell below 0 °C. The lower temperature tolerance of EGVM adults has not been established, but it is known from other moth species that both low temperatures and longer cold storage times can have negative impacts [30,31].

Many SIT programs have attempted to make releases during morning or evening hours to avoid releasing moths when temperatures are warm (G. Simmons personal observation, [32,33]), either by air release or by ground release directly in orchards or crops. High amounts of paved surfaces in urban environments may require adjustments to the release strategy to make releases earlier in the day or in the evenings after the heat of the day, which may improve moth performance and survivorship. Aerial release over gardens and trees may also be beneficial compared to ground release onto paved areas and may also increase moth survivorship, along with improved dispersion of moths, as seen in some other systems [34].

While a consistently high overflooding ratio was not maintained, there was a trend suggesting that sterile moth release may have had some control effect, as the peaks for the second and third flights were about 20 moths per trap/week lower in the release plot compared to the no-release plot (see Figure 7 and Figure 8). Despite failing to show a significant control effect, with the observation of early high overflooding ratios and a consistently high percentage of flyers in moth shipments, moth quality was reasonably high for much of the release period. This suggests that the production, shipping, and release system has the potential to deliver a high-quality moth to the field. It is unknown if more of a control effect would have been observed if moth releases were not halted for seven complete weeks and for three other occasions where only one release was made per week. More replication would be needed to more clearly demonstrate an effect of long-term releases.

The overflooding ratio needed to suppress wild EGVM is not known; however, if it is similar to results seen from several recent experimental and modeling studies that evaluated needed overflooding ratios for implementation of an IS strategy for moth SIT, a ratio between 5 and 15 may be sufficient to provide effective control [35,36,37,38,39,40,41]. However, determination of an effective overflooding ratio will be dependent on having a highly competitive moth [42,43,44,45]. Determination of needed overflooding ratios depends also on whether the program goal is suppression or eradication, with higher release ratios needed to ensure eradication outcomes, especially in the earlier stages of a program [28,38]. An important need going forward will be to directly test different sterile moth release rates in field cages or in small isolated plot studies to define needed overflooding ratios (see [35,36,39,45,46]). Monitoring mating using immobilized females moths on mating tables [16,47,48] or using light traps to capture wild females to examine for spermatophores marked by internal dye or stable isotopes [44,46,49] would provide additional means to evaluate male sterile moth mating success using different overflooding ratios.

With the sharp increase in wild moth populations seen during October through the end of February, it is clear that wild populations will need to be greatly reduced by other IPM measures in order to make use of an IS strategy as one of the main tools for sustainable control in these areas. There is a large population EGVM in this region, with average peaks as high as 80 moths per trap per week. The treated area of the release plot was relatively large for a field trial but may have not been isolated enough for the effect of release alone to control the population, as migration from other areas was likely significant, as shown by high populations in the nearby no-release plot. An analysis of the spatial distribution of wild moth captures for both the release and the control plots shows there was an uneven distribution of moth captures with a pattern of higher moth captures in the center of the plot relative to the edges (Figure S1, Supplementary Materials), suggesting that moth captures within these plots are local migrants rather than immigrants from surrounding crop land. However, there were three traps along the northern edge of the control field that corresponded to the highest captures of wild moths over the course of the experiment (Figure S1, Supplementary Materials). This local “hotspot” may show there is a large population of locally reproducing EGVM in that section of the city or that there are migrants coming from nearby grape production areas immediately adjacent to the Requinoa study area. It should be noted that the grape producers in this region are all participating in the area-wide control program, which includes the use of mating disruption and chemical treatments. The presence of these “hotspots” of wild moth captures on traps highlights the need to monitor spatial variation in EGVM population density to tailor sterile moth release rates to address this variation by increasing release rates in areas with higher density and not base release rates on the average wild moth density across the region [37,50,51].

This finding of wide variation in wild moth trap captures in the project area illustrates a need to develop GIS monitoring systems to provide timely data to SIT program mangers so that release rates and other control tactics can be adjusted to respond to changing conditions on the ground, which is a necessary feature common to successful area-wide control programs [50,51,52,53]. The SAG *Lobesia botrana* national control program has a critical piece of GIS infrastructure already in place with their development of an alert system built for monitoring and predicting EGVM populations and documenting control operation for growing areas [54]. Data on release and recapture rate of sterile moths also could be tracked in this manner and would aid the development of SIT as an operational control tool.

The releases in the project area were essentially a season-long mark–release–recapture study which can provide data for a common type of analysis used in ecological studies [55]. There are a number of studies, mainly from the mosquito SIT literature, that used this approach to estimate the wild population size within a control area to make estimates on needed release levels to achieve effective overflooding ratios (see [56,57,58]). It should be possible to use the monitoring data of sterile moth releases and wild moth populations to allow a similar approach to be used to make estimates of the size of regional EGVM populations.

In addition to using traps to monitor wild moth populations, estimating infestation densities for all life stages would add significant information for further development of the SIT and compatible IPM tools for control of EVGM in the urban landscape. Adding monitoring protocols to evaluate plant infestation levels and larval densities to the pilot project SIT evaluation would help to determine if the high wild moth population increase seen on monitoring traps is due to a locally reproducing population or due to migration from surrounding areas. This would help to target control activities and to determine the scale and scope of areas needing treatment by SIT. Observations of a decrease in larval and pupal infestation levels in the release area compared to no-release areas would provide compelling evidence that sterile moth releases are having an impact.

Successful deployment of an SIT strategy to control or suppress an established pest population requires combining several compatible IPM control measures on an area-wide basis to lower population levels enough to allow the release of sterile moths to be a cost-effective tactic [15,50,59,60,61]. A well-developed SIT control system can be a mainstay of a sustainable area-wide control program, but they are rarely successful as a standalone tactic without adoption of other control measures to decrease the size of the wild population [15,50,53,60]. Indeed, several moth SIT programs started first with a strategy of applying other area-wide control measures for several years to reduce the population size before introducing the use of SIT [15,53,62,63].

Challenges to manage outbreaks of invasive lepidoptera around the world are increasing and include the effects of climate change, expansion of global trade, and the arrival and establishment of new invasive species in urban areas close to agricultural production. It is these areas where conducting effective control programs can be difficult due to a lack of social acceptance [50,61]. Experience from eradication or suppression programs targeting pests that occupy these urban–agricultural areas have had mixed success. When programs have detailed pest population distribution and surveillance information, suitable control tools, and support by impacted stakeholders, i.e., both agricultural producers and residents, they are more likely to be successful [63,64,65,66,67]. The opposite result is possible [61,68,69], and this is why approaches that focus on softer and more socially acceptable approaches are needed [42,50,61,64].

Development of an effective SIT capacity will increase the likelihood of developing effective area-wide management of EGVM in the urban–agricultural interface zone when coordinated with regional producers’ treatments and combined with effective quarantine measures, the use of season long fruit stripping for grapevines and, where possible, applications of *Bacillus thuringiensis kurstaki* (Btk), and the use of hand-tied mating disruption dispensers. This kind of approach was successful in the treatment of urban areas to eradicate the painted apple moth in Auckland, New Zealand and included the use of SIT [63]. The successful EGVM eradication program in California, USA used a similar approach, and it had an element of good fortune whereby the infestation was discovered early when it was still small, but this program did not include the use of SIT [4,52,66].

## 5. Conclusions

Progress made to develop SIT capacity to control EGVM infesting urban areas adjacent to grape and fruit production areas includes the development of a mass-rearing system capable of producing 75,000 sterile moths per week, confirmation of 150 Gy as an adequate dose for implementation of an IS control strategy, and initiation of a season-long SIT release program in a section of a small city in central Chile. Because of a high wild moth population in this area, the releases of sterile moths did not reach large enough sterile to wild moth ratios to achieve effective control, but high moth quality and field performance were observed. Recommendations for further development of the SIT include conducting cage and field studies to evaluate overflooding ratios and mating competitiveness, measurement of larval infestation densities in release and no-release areas, and conducting trials to evaluate combining SIT with compatible IPM tactics such as fruit stripping and use of mating disruption.

## Figures and Tables

**Table 1:** Diet #46, one of the diets with the highest production of EGVM adults per unit of diet and sourced from ingredients available in Chile. Quantities for a 44 kg batch. All quantity units are in g.

Ingredient	Quantity	Percent
Tap water	28,600	65.0
Agar agar	572	1.3
Wheat bran	2640	6.0
Corn oil	44	0.1
Toasted soya flour	3036	6.9
Sugar	792	1.8
Wheat germ	2255	5.1
Nipagin	88	0.2
Potassium sorbate	88	0.2
Choline chloride	88	0.2
Formaldehyde (10% solution in water)	220	0.5
Acetic acid (25% solution in water)	748	1.7
Vitamínico, formula B ^1^	176	0.4
Sawdust	4664	10.6

^1^ Formula: calcium pantothenate (B-5) 25.38 g; amino nicotinic acid (B-3) 12.96 g; riboflavin 6.48 g; folic acid 6.48 g; hydrochloric thiamine (B-1) 3.24 g; hydrochloric pyroxydine (B-6) 3.24 g; biotin 0.26 g; B-12 0.016 g; formaldehyde (ca 37%) 0.5 mL; deionized water 1 μS 1000 mL; total wt. 1,058,556 g.

**Table 2:** Sterile EGVM male moth dispersal distances after capture on pheromone traps after point release experiments conducted from October 2018 to February 2019 (*n* = 48 traps/experiment).

Release No.	No. of Moths Released	No. Recaptured	% Recaptured	Mean (SD) Dispersal Distance (m)	Maximum Dispersal Distance (m)
1	2500	41	3.3%	50.5 (18)	102
2	5000	3	0.1%	36 (15.6)	58
3	5000	5	0.2%	86.4 (31.2)	133
4	5000	6	0.2%	71.2 (44.2)	155
5	5000	25	1.0%	66.5 (30)	133
Total	22,500	80	0.7%	58.8 (28.2)	155

**Table 3:** Mean (SD) percentage survival of irradiated mixed sex sterile EGVM held in small cages under field conditions during five replicate experiments conducted October 2018–February 2019. There were three cages of 250 moths per cage set up for each date.

Day	Mean % Surviving (SD)
1	90.0 (7.9)
2	76.8 (18.1)
3	61.5 (23.3)
4	34.4 (23.8)
5	19.9 (1.5)
6	NA
7	18.2 (20.9)
8	6.0 (7.8)
9	1.0 (2.0)
10	0

**Table 4:** Summary sterile EGVM release data for Requinoa pilot project conducted during August 2019–May 2020 (N/A = data not applicable).

Statistic	Number	Range	Comments
Release period	20 August 2019 to 5 May 2020	N/A	Two per week shipments by overnight air cargo from Arica
Total No. of shipments	57	N/A	17 missed releases: 1 missed release/week for 19 September 2019, 3 October 2019, 12 November 2019, and 12 December 2019; 7 complete missed weeks for 8 October to 29 October 2019, 11 January to 18 January 2020, and 7 April 2020
Shipping Temperatures	1.85–7.8 °C (average low, high)	−0.59–9.86 °C	Data available for 11 shipments
No. moths released/week, mean (SE)	20,334 (2705)	7107–60,430	
No. moths released/ha, mean (SE)	813 (108)	284–2417	
Total No. moths released	752,353	N/A	
Flyers/shipment (%), mean (SE)	80.9 (2.8)%	21.9–93.5%	Field laboratory flight testing at ambient outdoor temperatures

**Table 5:** Sterile male EGVM recapture data on pheromone traps in release and control fields. Requinoa pilot project, August 2019–May 2020.

Statistic	Number	Range	Comments
Total No. of sterile moths captured	3460	N/A	
Total No. of sterile moths captured in release plot	3374	N/A	97.5% of total recaptures
Total No. of sterile moths captured in no-release plot	86	N/A	2.5% of total recaptures
Recapture rate of male moths (%), mean (SE)	0.9 (0.1)%	0.1–2.2%	Calculated from previous week estimate of male moth release numbers
Overflooding ratio, mean (SE)	2.1 (1.2)	0–41.4	No. sterile/No. wild moths captured on traps

## Data Availability

Data is contained within the article and supplementary material.

## Supplementary Materials

The following are available online at https://www.mdpi.com/article/10.3390/insects12050378/s1: Figure S1. Spatial analysis of wild EGVM trap captures in release and control plots; Table S1: Adult EGVM parental and F_1_ sterility after 150 Gy treatment; Table S2: Adult EGVM parental sterility after 160 Gy treatment. Files S1–S6: All data used for analysis of irradiation studies and field trials.
