# Supplementary material for: Development of Sterile Insect Technique for Control of the European Grapevine Moth, Lobesia botrana, in Urban Areas of Chile
Source: Insects. 2021 Apr 22;12(5):378. doi: 10.3390/insects12050378 (PMC8143568; doi:10.3390/insects12050378)
Supplement: Supplementary file 1 [file insects-12-00378-s001.zip › Supplementary Material File 4-21-21/Supplementary Materials Table S1 4_16 Revised.pdf]

insects

MDPI

Review

Development of the sterile insect technique for the control of the European grapevine moth, *Lobesia botrana,* in urban areas of Chile next to grape and fruit production areas

Gregory. S. Simmons [1,*], Melissa Cristal Salazar Sepulveda [2,*], Edith Alejandra Fuentes Barrios [2,*], Marcela Idalsoaga Villegas [2], Raul Enrique Medina Jimenez [2], Alvaro Rodrigo Garrido Jerez [2,*], Ruth Henderson [1] and Hernán Donoso Riffo [2,3]

1 United States Department of Agriculture, Animal Plant Health Inspection Service, Plant Protection and Quarantine, Science and Technology, Salinas, CA, USA; Ruth.Henderson@usda.gov
2 Servicio Agrícola y Ganadero, Santiago and Arica, Chile; marcela.idalsoaga@sag.gob.cl; raul.medina@sag.gob.cl
3 Deceased
* Correspondence: Gregory.S.Simmons@usda.gov; Melissa.Salazar@sag.gob.cl; Edith.Fuentes@sag.gob.cl; Alvaro.Garrido@sag.gob.cl

Supplementary Material

Confirmatory Irradiation Studies

Methods

The experimental design for the evaluation of sterility for 150 Gy treatment for the parental (P_1) crosses was 3 replicate experiments, with 3 to 5 replicate crosses set up within each experiment for all the treated (T) by untreated (U) cross combinations along with a control treatment of U ♀ x U♂ crosses. For experiment 1, crosses were made with 10 mating pairs, in experiments 2 and 3, 20 mating pairs were used. Adult moths were collected from pupal emergence cages and placed into the 0.125 L plastic oviposition cages and supplied with sugar water on cotton wicks. These cages were maintained for 5 days at 22 ± 1° C, 65 ± 5% RH and 16:8 h LD. After each 24 h period, moths were moved to a new cage for a total of 5 separate collections of eggs. Egg strips were removed after 10 d to count the number of eclosed and uneclosed eggs.

To determine the level of F_1 sterility of irradiated males produced from the U♀ x T♂ cross, surviving eggs were infested on diet using the previously described larval rearing methods to rear to adult F_1 moths. Male and female F_1 adults obtained from these P_1 crosses were crossed with fertile adults from the opposite sex. Moths from the parental U♀ x U♂ cross were also reared to F_1 adults to use for a control cross for the F_1 sterility testing. Depending on the number of F_1 adults that were obtained, up to 5 replicate crosses for each sex were set up with as many as 20 mating pairs per cage. There were some replicates where far less material was available for 20 mating pairs per cage, data was collected only from crosses with at least 3 mating pairs. Eggs were collected and counted as described above.

The percentage sterility levels were determined by dividing the number of eclosed eggs by the total number of eggs x 100 and calculated for each day's egg collection for each cage. An index of sterility assessment was calculated using the method of Toppozada et al. 1966 for each category of cross. For analysis, the percentage of eggs eclosed were arcsine-square root transformed and the differences in the

number of eggs per female and percent egg hatch between each cross type were analyzed with one-way ANOVA, followed by a Tukey's HSD mean separation test where appropriate using the statistical software package JMP®, Version 13.1.0. (SAS Institute Inc., Cary, NC, 1989-2019).

Sterility data were also evaluated for 160 Gy treatment by setting up 4 replicate experiments with 4 replicate crosses for each experiment for all the irradiated cross combinations along with the control treatment of U♀ x U♂ crosses. Crosses were made with 10 mating pairs. Assays for sterility, calculations of the sterility index, and statistical analysis were performed as above. F_1 sterility was not assessed for these crosses.

Results

At 150 Gy there were no significant differences in the mean number of eggs produced per female for any of the irradiated cross treatments or the control ($F_{3,48}$ = 0.9041, p = 0.4461) (Table S1). There were significant differences in the percentage of egg hatch between the different cross types ($F_{3,48}$= 264.4, p < 0.001). The T♀ x T♂ and T♀ x U♂ crosses had the lowest percentage of egg hatch with means of 1.7% and 4.1% respectively compared to 53.1% for the U♀ x T♂ cross and 82.3% for the untreated control (Table S1).

For the F_1 crosses, there were significant differences for the mean number of eggs produced per female ($F_{2,99}$=22.7, p < 0.001) and for the mean percentage of egg hatch ($F_{2,99}$=212.6, p < 0.001) with 14.6%, 24.0%, and 83.9% for the U♀ x F_1♂, F_1♀ x U♂ and control cross respectively (Table S1).

The results for 160 Gy crosses, were similar in value to 150 Gy crosses for both the mean number of eggs per females which were not significantly different ($F_{3,60}$ = 1.2 , p = 0.326) and the mean percentage egg hatch which were significant ($F_{3,60}$ = 939 , p < 0.0001) (Table S2).

Table S1. Adult EGVM mean (SD) % egg hatch and % sterility index (IE) for P_1 crosses of treated (T) and untreated (U) moths and F_1 by U crosses after 150 Gy treatment. F_1 cross data shown are from the progeny of the parental cross between irradiated males and female moths (U♀ x T♂) . Numbers followed by different letters were significantly different by Tukey's HSD analysis.

Treatment	No. of Replicate Experiments	No. Mating Pairs per Experiment	Total No. Mating Pairs (♀ x ♂)*	No. Replicate Crosses per Experiment*	Mean (SD) No. Eggs per female	Mean (SD) % Egg Hatch	IE (%)
T♀ x U♂	3	20	230	5	80.2 (13.8)**a**	4.1 (2.4)**c**	95.1
U♀ x T♂	3	20	230	5	78.5 (13.6)**a**	53.1 (14.5)**b**	37.3
T♀ x T♂	3	20	230	5	76.4 (9.0)**a**	1.7 (1.5)**c**	98.1
U♀ x U♂	3	20	230	5	84.1 (12.1)**a**	82.3 (10.5)**a**	NA
**F_1 Crosses**							
F_1♀ x U♂	3	2-20	421	12-20	90.1 (27.2)**a**	23.9 (11.5)**b**	67.75
U♀ x F_1♂	3	5-20	646	12-20	58.3 (15.7)**b**	14.9 (11.3)**c**	80.16
U♀ x U♂	3	4-20	367	6-8	90.2 (28.4)**a**	83.9 (10.5)**a**	NA

*Experiment 1 had 10 mating pairs per cage and only 3 replicates.

Table S2. Adult EGVM mean (SD) % egg hatch and % sterility index (IE) for P_1 crosses after 160 Gy treatment. Numbers followed by different letters were significantly different by Tukey's HSD analysis.

Treatment	No. of Replicate Experiments	No. Mating Pairs per Experiment	Total No. Mating Pairs (♀ x ♂)	No. Replicate Crosses per Experiment*	Mean (SD) No. Eggs per female	Mean Egg Hatch (SD) (%)	IE (%)
T♀ x U♂	4	10	160	4	82.9 (13.9) **a**	3.4 (1.9) **c**	96.2
U♀ x T♂	4	10	160	4	84.9 (13.8) **a**	65.1 (8.7) **b**	25.5
T♀ x T♂	4	10	160	4	75.0 (18.3) **a**	1.3 (1.1) **d**	98.7
U♀ x U♂	4	10	160	4	82.7 (17.6) **a**	89.7 (4.8) **a**	NA

Spatial Analysis of Wild EGVM Trap Captures in Release and Control Plots

To help analyze the distribution of the wild EGVM population in the city of Requinoa and a possible source for the migration of moths into the release plot, the mean wild moth catch per trap per week was calculated by location for all the traps in the release and control plots. These were plotted on a map of the city showing trap catch in each location using a relative heat map scale (Figure S1). We evaluated the hypothesis of an equal distribution of moths within each plot for the release and control plots plot by one-way ANOVA after the Box-Cox transformation to normalize a data set with many 0s. The mean (SD) number of wild EGVM caught per trap per week ranged from 0.9 (1.7) to 35.7 (42.2) in the release plot and from 1.4 (2.3) to 132.5 (62.7) in the control plot and differed significantly among traps within plots ($F_{33,1107}$ =4.95, p<0.001) and ($F_{27,883,}$ =3.94, p<0.001) respectively. The statistical software package JMP®, Version 13.1.0. was used for this analysis (SAS Institute Inc., Cary, NC, 1989-2019).

.

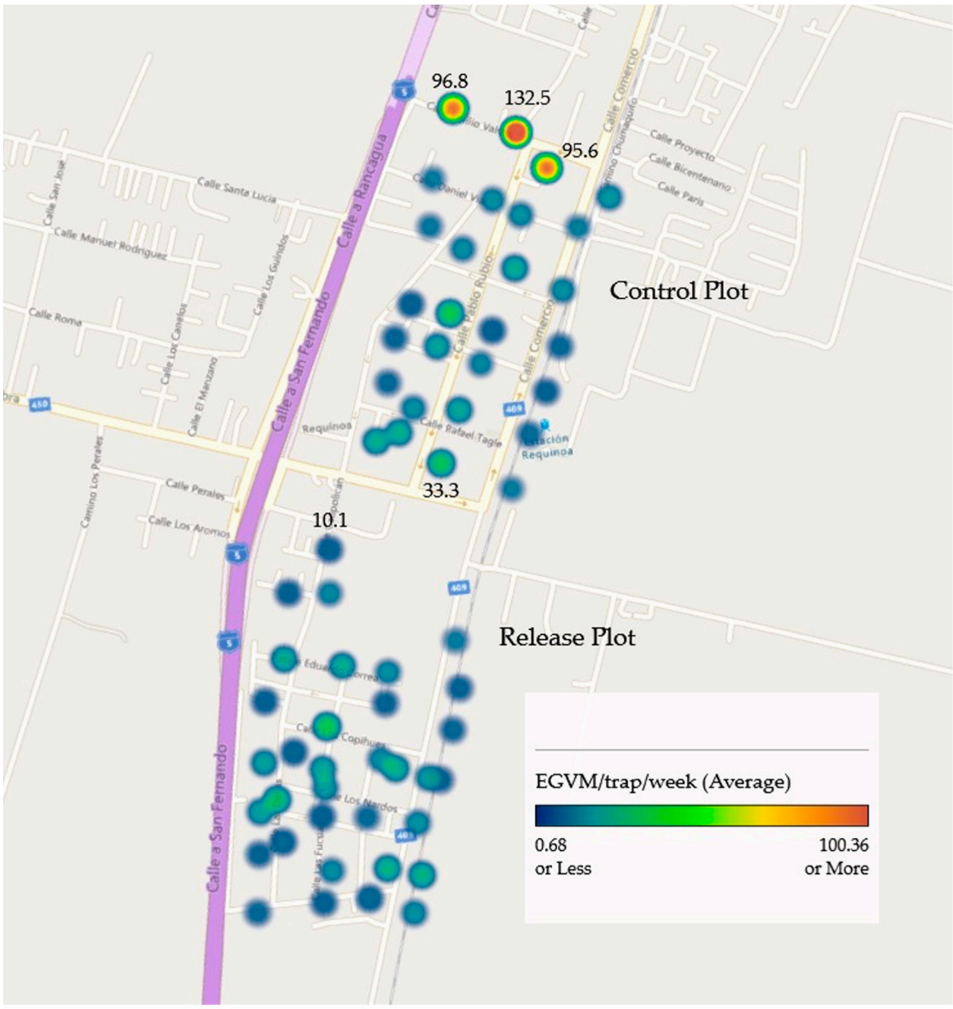

Figure S1. Distribution of the mean number of wild EGVM moth captures per trap over the course of the season for both the release and control plots in the city of Requinoa during the experiment conducted between August 2019 to May 2020. The mean number of EGVM caught per trap marked on some traps to help calibrate the relative heat map scale. Within each plot the mean number of wild moths caught per week differed significantly among traps ($F_{33,1107}$ =4.95, p<0.001) and ($F_{27,883,}$ =3.94, p<0.001) respectively. Release field N = 34 traps, control field N = 28 traps.

References

Toppozada, A.; S. Abdallah; M. Eldefrawi. Chemosterilization of larvae and adults of the Egyptian cotton leafworm *Prodenia litura* , by apholate, metepa, and tepa . *J. Econ. Entomol.*, **1966**. **59**: p. 1125 – 1128 .
